# Amelogenesis Imperfecta or Molar-Incisor Hypomineralisation: A Narrative Review and Diagnostic Framework for Differentiating a Genetic From an Environmental Enamel Defect in Children

**DOI:** 10.7759/cureus.112445

**Published:** 2026-07-11

**Authors:** Samir Laouina

**Affiliations:** 1 Provincial Directorate of Mohammedia, Regional Academy of Education and Training of the Casablanca–Settat Region, Ministry of National Education, Mohammedia, MAR

**Keywords:** amelogenesis imperfecta, developmental enamel defects, differential diagnosis, genetic testing, molar-incisor hypomineralization, pediatric dentistry

## Abstract

Amelogenesis imperfecta (AI) and molar-incisor hypomineralization (MIH) are two developmental enamel defects of distinct etiologies that may present overlapping clinical features, particularly when AI manifests as hypocalcified or hypomature phenotypes. This overlap constitutes a well-recognized diagnostic challenge at all levels of dental education and has important implications for treatment planning.

This narrative review aims to synthesize the available data on the clinical, radiographic, genetic, and epidemiological features that allow differentiation between AI and MIH in children and to propose a stepwise diagnostic framework to support accurate and timely clinical decision-making. A narrative review was conducted using the PubMed database, supplemented by Google Scholar. The search covered the period from January 2000 to April 2026. Studies were included if they reported clinically relevant characteristics of AI and/or MIH with sufficient detail to support differential diagnosis. A total of 16 studies were selected for the narrative synthesis. Generalized, symmetric enamel abnormalities affecting both dentitions are suggestive of AI, whereas asymmetrical, localized opacities primarily involving permanent first molars and incisors are more indicative of MIH. Taurodontism was reported more frequently in AI than in MIH. Diagnostic confusion between the two conditions is well documented in surveys of general dental practitioners, dental students, and in image-based classification models. Family history, syndromic manifestations, and next-generation sequencing support the diagnosis of AI when it is clinically suspected.

Differentiating between AI and MIH requires a structured clinical assessment integrating lesion distribution, symmetry, involvement of dentitions, family history, radiographic findings, syndromic screening, and targeted genetic testing. The proposed 10-step diagnostic framework aims to reduce diagnostic uncertainty and guide referral for specialized management in pediatric patients presenting with enamel defects of uncertain etiology.

## Introduction and background

Developmental defects of enamel are a heterogeneous group of structural and mineralization abnormalities that arise during odontogenesis as a result of complex interactions among genetic, epigenetic, and environmental factors [1,2]. In children, these anomalies may affect the quantity, quality, or both aspects of enamel, depending on the timing and nature of the disturbance in ameloblastic function [3]. Clinically, they may result in hypersensitivity, increased susceptibility to caries, aesthetic concerns, post-eruptive breakdown, and complex long-term treatment needs [4,5]. An accurate diagnosis is therefore essential not only for disease classification but also for appropriate management, whether preventive, restorative, orthodontic, or multidisciplinary. Amelogenesis imperfecta (AI) is a hereditary disorder of enamel formation, characterized by generalized enamel abnormalities that typically affect all teeth in a relatively symmetrical pattern and may involve both the primary and permanent dentitions [4,6]. It is clinically and genetically heterogeneous, with pathogenic variants described in several genes involved in enamel matrix formation and mineralization, including AMELX, ENAM, MMP20, KLK4, and DLX3 [7]. AI may be inherited in X-linked, autosomal dominant, or autosomal recessive patterns, and its reported prevalence varies widely, from approximately 1:700 to 1:14,000 [4]. AI phenotypes are commonly classified as hypoplastic (reduced enamel thickness), hypomature (normal enamel thickness with impaired maturation resulting in softer enamel), hypocalcified (normal enamel thickness with severe mineralization defects leading to poorly calcified enamel), or mixed forms, including hypomaturation-hypoplasia with taurodontism variants [4,6].

Molar-incisor hypomineralization (MIH), by contrast, is generally considered a qualitative defect of enamel mineralization of multifactorial origin. It primarily affects one or more permanent first molars, with or without involvement of the permanent incisors, and typically presents as demarcated opacities that may progress to post-eruptive enamel breakdown [8,9]. Unlike AI, MIH does not follow a clearly established Mendelian pattern of inheritance and is generally more localized and asymmetrical in presentation [8,10]. Its reported prevalence is considerably higher than that of AI, with global estimates ranging from approximately 10% to 25% and an average prevalence of about 13.5% in recent reviews [3,9]. Hypomineralized second primary molars have also been described as a predictive factor for MIH, supporting the concept of a broader spectrum of hypomineralization affecting both primary and permanent dentitions [10].

Despite these conceptual differences, distinguishing between AI and MIH may be clinically challenging. Hypocalcified and hypomature forms of AI may present with hypomineralized enamel, demarcated opacities, hypersensitivity, and post-eruptive breakdown, thereby resembling MIH during routine clinical examination [5,11]. This overlap has also been documented in the published literature. In a survey conducted among 311 dental practitioners, 38.7% reported difficulty differentiating MIH from AI, whereas this difficulty was reported by only 9.8% of pediatric dentists, highlighting the importance of clinical experience and specialized training [12]. Similar clinical challenges have been reported among dental students and general practitioners, with AI and MIH frequently being mistaken for one another during clinical assessment [13,14]. Image-based artificial intelligence studies further confirm the existence of substantial visual overlap, with AI reported as one of the enamel defects most difficult to classify accurately using convolutional neural networks [15].

The distinction between AI and MIH has important clinical implications. AI generally requires long-term planning involving the entire dentition, including preventive care, restorative rehabilitation, orthodontic considerations, and, in some cases, genetic counseling or syndromic evaluation. Management of MIH is typically focused on the affected permanent first molars and incisors, although severity may vary considerably [4,5]. Misdiagnosing AI as MIH may therefore lead to underestimation of disease extent, inappropriate extraction decisions, delays in multidisciplinary management, and failure to recognize syndromic forms of AI. Conversely, misclassifying MIH as AI may expose patients to unnecessary genetic concerns and overly complex treatment planning. Additional clinical and radiographic features, such as family history, involvement of both dentitions, taurodontism, eruption disturbances, anterior open bite, and systemic manifestations, may support the diagnostic approach but must be interpreted within a structured clinical assessment framework [16-18].

Although several studies and reviews have described the clinical characteristics of AI and MIH, the available data remain dispersed across diagnostic surveys, clinical reviews, genetic studies, radiographic analyses, and expert recommendations. A practical framework integrating distribution, symmetry, dentition involvement, family history, enamel phenotype, radiographic findings, orthodontic features, syndromic signs, and genetic testing could assist clinicians in approaching enamel defects of uncertain origin in a more systematic manner. Therefore, the present narrative review aims to synthesize the available evidence on the differentiation between AI and MIH in children and to propose a stepwise diagnostic framework to support accurate and timely clinical decision-making.

Methods

Review Design

This study was conducted as a narrative (non-systematic) review. A narrative approach was selected because of the heterogeneity of the available literature addressing the distinction between amelogenesis imperfecta (AI) and molar-incisor hypomineralization (MIH), which includes case series, descriptive cross-sectional studies, retrospective analyses, and general reviews on enamel defects that do not specifically focus on this diagnostic distinction.

Given this heterogeneity and the predominantly descriptive nature of the available data, a formal systematic review with quantitative synthesis would not adequately address the clinical objective of the present study. A narrative synthesis was therefore considered more appropriate for integrating dispersed clinical, radiographic, and phenotypic observations into a practical and operational diagnostic framework.

The primary objective of this review was to synthesize clinically relevant evidence in order to support differential diagnosis and to develop a structured and gradual stepwise diagnostic framework applicable to clinical practice.

Search Strategy

A bibliographic search was conducted using PubMed (MEDLINE) as the primary database. A supplementary search was performed in Google Scholar to identify additional peer-reviewed articles not indexed in PubMed, including regional publications.

The search covered the period from January 2000 to April 2026, with particular attention given to recent studies published between 2020 and 2026 to ensure up-to-date bibliographic coverage. The final search was conducted on April 30, 2026. Following peer review, the literature search was updated in July 2026 to identify newly published studies. One additional relevant systematic review was identified and cited in the Discussion to reflect the most recent evidence.

The following search strategies were used:

Core search (AI and MIH): ("Amelogenesis Imperfecta"[Mesh] OR "amelogenesis imperfecta"[tiab] OR AI[tiab]) AND ("Molar Incisor Hypomineralization"[Mesh] OR "molar incisor hypomineralization"[tiab] OR MIH[tiab])

Second, a differential diagnosis refinement was applied by combining the core search with ("differential diagnosis"[Mesh] OR "clinical differentiation"[tiab] OR "misdiagnos*"[tiab])

Third, a clinical features supplement was added using the query: ("amelogenesis imperfecta"[tiab] OR "molar incisor hypomineralization"[tiab]) AND ("clinical features"[tiab] OR "phenotype"[tiab])

No restrictions regarding the study design were applied in order to ensure a comprehensive identification of clinically relevant descriptive data. The reference lists of the included articles were manually screened to identify any additional relevant publications.

**Figure 1:**
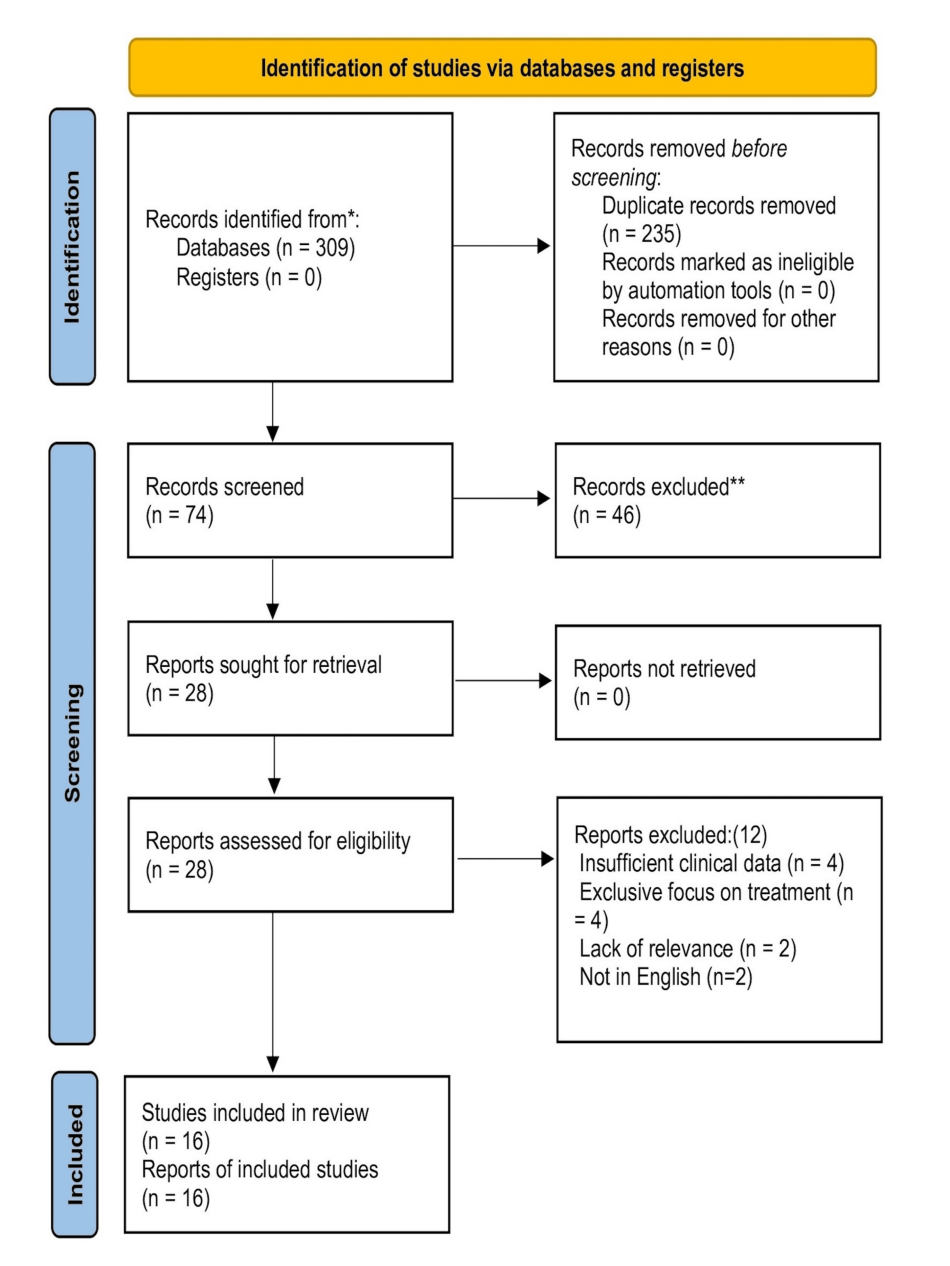
Flowchart of the study selection process Records identified through database searching (n=309) were screened after title and abstract review (n=74). Full-text articles were assessed for eligibility (n=28), and 16 studies were included in the final narrative synthesis.

To enhance transparency in reporting the literature selection process, a Preferred Reporting Items for Systematic Reviews and Meta-Analyses (PRISMA) 2020-style flow diagram was used to document study identification, screening, eligibility assessment, and inclusion. The initial database search identified 309 records. After removing duplicates, 74 records remained for title and abstract screening. Of these, 46 records were excluded based on the eligibility criteria, leaving 28 full-text articles for detailed assessment. Following full-text review, 12 articles were excluded for reasons including insufficient clinical data, exclusive focus on treatment without diagnostic differentiation, or lack of relevance to the AI-MIH distinction. A total of 16 studies met the inclusion criteria and were included in the final narrative synthesis. The study selection process is summarized in Figure 1.

Inclusion and Exclusion Criteria

*Inclusion criteria: *Studies were included if they met the following criteria: original clinical studies reporting the characteristics of AI and/or MIH with sufficient detail to allow the extraction of discriminative clinical features; studies providing direct or indirect comparisons between AI and MIH; review articles addressing the differential diagnosis or clinical overlap among enamel defects (included for contextual synthesis purposes); case series involving at least five patients; studies involving pediatric patients with AI and/or MIH, or studies addressing the diagnosis, differential diagnosis, clinical characteristics, genetics, radiographic features, or professional recognition of these conditions; and articles published in English.

*Exclusion criteria*: The exclusion criteria were as follows: animal or in vitro studies without clinical correlation; isolated case reports (except those illustrating diagnostic confusion, which were included for qualitative purposes only); studies focusing exclusively on treatment, molecular genetics without clinical phenotypic characterization, or laboratory biomarkers; conference abstracts for which no full-text article was available; and publications in languages other than English.

*Data extraction and synthesis*: Data extraction was performed by the sole author using a predefined data extraction form. To minimize selection and extraction bias, the extracted data were cross-checked against the full text of each included article prior to the synthesis phase. For each eligible study, data were extracted into a standardized table including first author, publication year, and country; study design and sample size; diagnostic criteria used (e.g., Witkop classification for AI; EAPD criteria for MIH); clinical characteristics (symmetry, distribution, affected dentition, enamel appearance, and post-eruptive breakdown); radiographic findings (enamel thickness, taurodontism, and associated dental anomalies); family history (reported or absent); associated systemic or extraoral manifestations; and any explicit statements regarding the distinction between AI and MIH.

Features were considered discriminative when they were consistently reported across multiple studies or explicitly identified by the authors as relevant to the differential diagnosis. Divergent or overlapping findings were retained and analyzed qualitatively to reflect the diagnostic uncertainty encountered in real-world clinical practice.

The extracted data were synthesized into a comparative table highlighting the distinguishing features of AI and MIH. The 10-step diagnostic framework was developed by integrating the diagnostic indicators most consistently reported across the included studies and organizing them according to their sequential clinical applicability. Based on this synthesis, a stepwise diagnostic framework was developed, focusing on key discriminative domains, including symmetry, distribution, family history, and structural enamel characteristics.

This framework was refined iteratively to ensure consistency with the overall body of evidence identified in the included literature. No major findings that would invalidate the proposed framework were identified, although certain variations and exceptions have been reported in the literature.

Limitations of the narrative approach

This review has several limitations inherent to its narrative design. The search strategy did not include multiple databases such as Embase or Scopus; however, supplementary searches were conducted to minimize the risk of overlooking relevant studies. No formal risk-of-bias assessment was performed, and the included studies exhibited variable methodological quality. The proposed diagnostic framework was derived from published evidence and has not been prospectively validated in a clinical setting. Publication bias cannot be excluded, as studies reporting clear distinctions between conditions are likely to be published more frequently than those highlighting diagnostic overlap or ambiguity. As data extraction and synthesis were conducted by a single reviewer, the possibility of selection and interpretation bias cannot be entirely excluded.

Despite these limitations, the narrative synthesis allows for the integration of heterogeneous clinical data into a practical framework aimed at supporting diagnostic decision-making in paediatric dentistry.

Ethics statement

As this study was based exclusively on previously published data, no ethical approval was required. No formal review protocol was registered due to the narrative and exploratory nature of this review.

## Review

Results

A total of 309 references were initially identified through the literature search process. After screening titles, abstracts, and full texts according to the eligibility criteria, 16 articles were included in the final narrative synthesis.

The included studies exhibited heterogeneous designs, comprising cross-sectional surveys, narrative reviews, scoping reviews, systematic reviews, retrospective analyses, comparative observational studies, consensus guideline documents, molecular genetic investigations, and diagnostic imaging studies. Overall, the reviewed literature addressed diagnostic confusion between AI and MIH, as well as the clinical, radiographic, orthodontic, genetic, syndromic, and therapeutic characteristics contributing to their differential diagnosis. The main features and diagnostic contributions of the included studies are summarized in Table 1.

**Table 1:** Characteristics and diagnostic contribution of included studies AI: Amelogenesis imperfecta; MIH: molar-incisor hypomineralization; EAPD: European Academy of Paediatric Dentistry; HSPM: hypomineralised second primary molar; GDP: general dental practitioner; NGS: next-generation sequencing.

First author, year	Study design	Population/sample	Main focus	Main contribution for AI–MIH differentiation
Marquillier et al. (2025) [12]	Cross-sectional survey	311 dentists	Diagnostic confusion	A total of 38.7% of general dental practitioners reported difficulty in differentiating AI from MIH, whereas this difficulty was less frequently reported by paediatric dentists.
Bin Saleh (2023) [4]	Scoping review	33 studies	AI clinical characteristics	Prevalence of AI, phenotypic classification, multidisciplinary management, differential diagnosis.
Lakhani et al. (2025) [5]	Consensus guidance	Expert panel	Clinical management	Hypocalcified and hypomature AI can mimic MIH, underscoring the importance of early differentiation.
Broutin et al. (2023) [17]	Systematic review	339 AI patients	Orthodontic findings	Open bite was the most prevalent malocclusion in AI (27.7%), with higher prevalence in hypoplastic (35.5%) and hypomineralized (36.7%) phenotypes. No genotype-phenotype correlation could be established.
Smith et al. (2024) [18]	Molecular case series	8 AI patients	Syndromic AI	AI associated with sensorineural hearing loss and syndromic manifestations.
Hu et al. (2007) [7]	Narrative review	Published mutation data	AI genetics	Identification of the main genes involved in AI, including AMELX, ENAM, MMP20, KLK4, and DLX3.
Neves et al. (2015) [3]	Clinical review	MIH case + literature	MIH characteristics	MIH is a qualitative enamel defect; HSPM are predictive of MIH.
Jianu et al. (2022) [9]	Narrative review	40 articles	Differential diagnosis	AI is typically generalized and hereditary, whereas MIH is usually localized and asymmetrical.
Möhlhenrich et al. (2024) [11]	Retrospective study	19 AI patients	Orthodontic phenotype	Anterior open bite and eruption disturbances reported in AI.
Alevizakos et al. (2022) [15]	CNN diagnostic study	462 clinical images	AI image classification	AI shows the greatest phenotypic overlap with other enamel defects.
Cagetti et al. (2022)[13]	Questionnaire survey	301 students	Diagnostic knowledge	Frequent confusion between AI and MIH among students.
Humphreys et al. (2021) [14]	Diagnostic vignette survey	76 GDPs	MIH diagnosis	AI is frequently among the misdiagnoses proposed for MIH.
Dulla and Meyer-Lueckel (2021) [8]	Narrative review	Review	Clinical differentiation	Distribution and symmetry are identified as the main distinguishing criteria.
Bandeira Lopes et al. (2026) [10]	Scoping review	57 studies	MIH/HSPM spectrum	MIH asymmetry is emphasized; HSPM are predictive of MIH.
Casas Araya et al. (2021) [16]	Comparative observational study	12 MIH vs 10 AI	Radiographic comparison	Taurodontism is significantly more frequent in AI.
Bloch-Zupan et al. (2023) [6]	Retrospective cohort	115 AI index cases	NGS diagnosis	Diagnostic yield of 60%; characterization of syndromic and non-syndromic AI.

Clinical challenges in distinguishing AI from MIH

Several studies have reported substantial difficulty in distinguishing AI from MIH, particularly among non-specialist practitioners and students. In a cross-sectional survey involving 311 dental practitioners, Marquillier et al. [12] reported that 64% of participants experienced difficulty distinguishing MIH from other enamel defects, with 38.7% specifically struggling to differentiate it from AI. This difficulty was markedly less pronounced among pediatric dentists, suggesting an experience-related gradient in diagnostic performance.

Cagetti et al. [13] also highlighted bidirectional diagnostic confusion among dental and dental hygiene students. AI was correctly identified as a genetic disorder by 64.45% of the respondents, whereas MIH was correctly identified from clinical images by only 52.81%. Similarly, Humphreys et al. [14] observed that general dental practitioners frequently included AI among the incorrect differential diagnoses proposed for MIH clinical vignettes, with no significant difference in diagnostic accuracy between practitioners with and without postgraduate qualifications.

This diagnostic overlap was further supported by the imaging-based study conducted by Alevizakos et al. [15], which employed convolutional neural networks to classify enamel defects. AI proved to be the most challenging category to classify accurately, with the VGG16 network achieving an accuracy of only 54.76% for AI, representing the lowest performance among the conditions evaluated. Taken together, these findings suggest that AI-MIH diagnostic confusion reflects both limited diagnostic familiarity and genuine visual overlap between hypomineralized enamel defects, rather than being attributable solely to deficiencies in clinical training [12,15].

Distribution patterns and enamel phenotypic characteristics

The distribution and symmetry of enamel defects were the most consistently reported clinical indicators for differentiating AI from MIH. Dulla and Meyer-Lueckel [8] described AI as a generalized and symmetrical condition affecting the entire dentition, whereas MIH primarily involves the first permanent molars, with or without incisor involvement, and frequently presents asymmetrically. Bandeira Lopes et al. [10] further reinforced this distinction by reporting that MIH lesions are asymmetrical in up to two-thirds of cases, whereas AI is typically characterized by generalized and symmetrical involvement.

The pattern of dentition affected represented another recurrent differential diagnostic criterion. Jianu et al. [9] reported that AI may affect both the primary and permanent dentitions, whereas MIH predominantly affects the permanent dentition and is characterized by well-demarcated opacities. Neves et al. [3] further emphasized that MIH is a qualitative enamel defect resulting from hypomineralization, whereas enamel hypoplasia represents a quantitative defect in enamel formation. The same study also identified hypomineralized second primary molars as a predictive factor for MIH.

Epidemiological data further contextualize the diagnostic challenge. The prevalence of AI has been reported to range from approximately 1:700 to 1:14,000, whereas MIH is estimated to affect approximately 10%-25% of children worldwide [3,4,9].

Despite the diagnostic value of distribution and symmetry, several studies have reported substantial clinical overlap between the hypomineralized phenotypes of AI and MIH. Lakhani et al. [5] reported that hypocalcified and hypomature forms of AI may resemble MIH because of shared hypomineralization features and overlapping clinical presentations. Similarly, Möhlhenrich et al. [11] noted that hypomineralized AI can clinically mimic MIH during routine examination.

Radiographic, orthodontic, and skeletal findings

Radiographic and craniofacial data were more frequently associated with AI than with MIH and may provide additional information in the differential diagnosis. In a comparative observational study, Casas Araya et al. [16] reported taurodontism in 60% of patients with AI compared with 16.7% of those with MIH, and apical dilaceration in 90% versus 16.7%, respectively [16]. The same study also identified differences in the skeletal profile between the two groups. However, these comparative findings should be interpreted with caution due to the limited sample size [16].

Taurodontism has also been reported in association with specific AI phenotypes and genotypes. Bloch-Zupan et al. [6] identified it in association with several pathogenic variants related to AI, particularly type IV AI and DLX3-related forms. Nevertheless, Bloch-Zupan et al. [6] also reported that type IV AI represented only a small proportion of their cohort, suggesting that taurodontism may be diagnostically specific when present, but insufficiently sensitive to exclude AI when absent [6].

Orthodontic and skeletal manifestations have also been described in AI. Broutin et al. [17] reported anterior open bite in 35.5% of hypoplastic AI cases and 36.7% of hypomineralized AI cases, with particularly high prevalence in cases associated with FAM20A mutations. Similarly, Möhlhenrich et al. [11]. described open bite, eruption disturbances, and dolichofacial tendencies in patients with AI. Overall, these findings indicate that radiographic and orthodontic signs may strengthen the suspicion of AI, although none of these features is individually pathognomonic [11].

Genetic and syndromic considerations

The genetic context has consistently been identified as a major distinguishing factor between AI and MIH. Hu et al. [7] described several genes involved in the pathogenesis of AI, including AMELX, ENAM, MMP20, KLK4, and DLX3, thereby confirming the hereditary nature of AI. In contrast, MIH has generally been described in the reviewed literature as a multifactorial enamel defect that does not follow a clearly defined Mendelian inheritance pattern [8,9].

The role of molecular diagnosis was further highlighted by Bloch-Zupan et al. [6], who applied next-generation sequencing to 115 AI index cases and reported an overall diagnostic yield of approximately 60%. Within this cohort, 81% of index cases carried likely pathogenic or pathogenic variants, with non-syndromic AI accounting for 73% of cases and syndromic AI for 27%. The authors also identified several previously unreported variants, underscoring the genetic heterogeneity of AI [6].

Syndromic forms of AI were also represented in the reviewed literature. Smith et al. [18] described an autosomal recessive syndrome linked to PLXNB2, characterized by AI, sensorineural hearing loss, intellectual disability, lymphoedema, and ocular involvement. The authors explicitly noted that this phenotype may be clinically misinterpreted as MIH or fluorosis [18]. Bin Saleh [4] further emphasized the importance of early diagnosis and multidisciplinary management of AI.

Collectively, these studies highlight the importance of family history, systemic manifestations, and genetic evaluation when AI is suspected, particularly in generalized, symmetrical, familial, or syndromic presentations.

Therapeutic implications

Several studies have emphasized that early and accurate differentiation between AI and MIH has direct implications for clinical management. Lakhani et al. [5] highlighted that treatment planning differs substantially between AI and MIH, as the generalized involvement observed in AI may affect both erupted and unerupted teeth. In particular, decisions regarding interceptive extractions in AI must be made with caution due to the global nature of the dental involvement, whereas treatment decisions in MIH are typically limited to the affected first permanent molars and incisors.

Bin Saleh [4] further reported that no standardized therapeutic protocol currently exists for AI, and that multidisciplinary management and early diagnosis are recurrent recommendations in the literature. These findings indicate that diagnostic accuracy is crucial not only for disease classification, but also for restorative planning, orthodontic decision-making, patient counselling, and appropriate specialist referral [4].

Overall, the included studies support a structured diagnostic approach combining clinical examination, assessment of lesion distribution and symmetry, family history, radiographic evaluation, orthodontic findings, systemic assessment, and genetic testing when indicated.

Discussion

Why AI and MIH Are Frequently Confused Diagnostically?

This review highlights that the distinction between AI and MIH remains clinically challenging, particularly when AI occurs in its hypomineralized forms. In such cases, both conditions may share similar visible features, including enamel opacities, hypersensitivity, and post-eruptive breakdown. Consequently, visual inspection alone may be insufficient, especially when clinicians focus on the most severely affected teeth without systematically evaluating the entire dentition.

Diagnostic confusion appears to be primarily driven by three factors: phenotypic overlap between hypocalcified or hypomature forms of AI and MIH; greater clinical familiarity with MIH compared with rare forms of AI; and the absence of a systematic diagnostic approach incorporating lesion distribution, symmetry, dentition involvement, family history, and radiographic findings. Available evidence also suggests that this difficulty is not solely attributable to insufficient training but also reflects a genuine visual overlap between certain hypomineralized enamel defects. This observation supports the need for a structured diagnostic framework rather than an approach based exclusively on visual lesion recognition [12-15].

Clinical Value and Limitations of Major Diagnostic Indicators

Distribution and symmetry should be considered the first diagnostic step when AI and MIH are suspected [8,9,16]. A localized and asymmetrical pattern involving the first permanent molars, with or without the incisors, is suggestive of MIH. In contrast, generalized and symmetrical enamel involvement, particularly affecting both arches and multiple tooth groups, should raise suspicion of AI.

Dentition involvement provides an additional diagnostic clue. AI may affect both primary and permanent dentitions, whereas MIH predominantly involves the first permanent molars and incisors, although the presence of hypomineralized second primary molars may indicate an increased risk of MIH. Clinical history regarding the primary dentition, as well as examination of retained primary teeth, may therefore be useful, particularly in the mixed dentition stage [3,10].

These clinical criteria should not, however, be interpreted in isolation. Some definitions of MIH and recent reviews emphasize that MIH may involve teeth beyond the classically described first permanent molars and incisors. Conversely, early AI may be difficult to recognize before full eruption of the permanent dentition. For this reason, distribution and symmetry are strong clinical indicators but not definitive diagnostic criteria [5,11].

Radiographic findings may provide valuable complementary information, particularly when clinical examination is inconclusive. Taurodontism was the most consistently reported radiographic feature associated with AI and was significantly more frequent in AI than in MIH in the only comparative study identified. Its presence, particularly in a patient with generalized enamel defects, should therefore strengthen the suspicion of AI [6,16].

However, taurodontism must be interpreted with caution. It is not present in all cases of AI and appears to be more closely associated with specific phenotypes or genotypes, particularly type IV AI and DLX3-related forms. Its limited sensitivity should be emphasized, as type IV AI represented only a small proportion of the cohort reported by Bloch-Zupan et al. [6]. Accordingly, taurodontism may have relatively high diagnostic value when present, but its absence does not exclude AI.

Orthodontic and skeletal features, including anterior open bite, eruption disturbances, and dolichofacial tendencies, have also been reported in AI cohorts. These characteristics may assist specialists in identifying broader craniofacial involvement, but they are not pathognomonic. Their main value is therefore contributory rather than diagnostic. In clinical practice, radiographic and orthodontic assessments should be used to strengthen or weaken diagnostic suspicion rather than replace clinical recognition of enamel phenotypes [11,17].

The genetic basis of AI remains one of the most important distinctions from MIH [6,7]. AI is a hereditary enamel disorder associated with multiple genes involved in enamel formation, whereas MIH is generally considered multifactorial and does not follow a clearly defined Mendelian inheritance pattern [8,9]. A positive family history, particularly when multiple family members present with similar generalized enamel defects, strongly supports a diagnosis of AI [6,7].

Molecular studies further highlight the growing clinical role of next-generation sequencing. Genetic testing can confirm AI, identify the underlying genotype, distinguish syndromic from non-syndromic forms, and guide family genetic counselling [6]. However, genetic testing should not be considered a first-line investigation for all enamel defects. Its use is most appropriate when clinical features are suggestive of AI, particularly in generalized, symmetrical, familial, severe, or syndromic presentations [4,6,18].

Syndromic manifestations are of particular importance. AI associated with sensorineural hearing loss, ocular anomalies, lymphoedema, developmental disorders, or other systemic features should prompt referral for specialist evaluation and potential genetic investigation. In such cases, misdiagnosing AI as MIH may delay recognition of broader systemic conditions [18].

Clinical Implications of Accurate Differentiation

Early and accurate differentiation between AI and MIH is clinically important, as these two conditions differ not only in their etiology but also in their therapeutic management [4,5]. The management of MIH is generally focused on the affected first permanent molars and incisors, whereas AI often requires long-term planning involving the entire dentition [4,8]. This distinction has implications for preventive care, restorative decision-making, orthodontic planning, prosthodontic rehabilitation, and patient counselling [4,5].

This distinction is particularly relevant when interceptive extractions are considered. In MIH, extraction decisions are generally limited to severely affected molars. In contrast, in AI, the generalized nature of the condition means that treatment decisions may influence overall occlusion and the future management of both erupted and unerupted teeth. Therefore, suspected cases of AI should ideally be evaluated by specialists in paediatric dentistry, orthodontics, and restorative dentistry prior to any irreversible therapeutic decisions [4,5].

Proposed Stepwise Diagnostic Framework

Based on the recurrent diagnostic indicators identified in the reviewed studies [8-11,16], a structured and stepwise approach may assist clinicians in differentiating AI from MIH in routine practice. This framework is not intended to replace specialist diagnosis or genetic testing, but rather to guide clinical reasoning and identify cases requiring referral. The main steps and clinical interpretations of the proposed diagnostic framework are summarized in Table 2.

**Table 2:** Stepwise diagnostic framework for differentiating AI from MIH AI: Amelogenesis imperfecta; MIH: molar-incisor hypomineralization; HSPM: hypomineralised second primary molar

Step	Diagnostic domain	Evidence supporting AI	Evidence supporting MIH	Clinical interpretation
1	Distribution	Generalized enamel defects affecting multiple teeth or the entire dentition	Primarily permanent first molars ± incisors	Primary discriminator, highly useful
2	Symmetry	Symmetric involvement	Asymmetric involvement	Strong clinical indicator
3	Affected dentitions	Primary and permanent dentitions may be affected	Mainly permanent dentition; HSPM may predict MIH	Useful in mixed dentition
4	Enamel phenotype	Hypoplastic, hypomature, hypocalcified, or mixed defects	Demarcated opacities, post-eruptive breakdown, qualitative hypomineralization	Possible overlap in hypomineralized AI
5	Family history	Positive family history or Mendelian pattern	Usually absent or non-specific	Strongly suggestive of AI when present
6	Radiography	Taurodontism, apical dilaceration, eruption disturbances	Usually absent or poorly characterized	Indicative, non-definitive findings
7	Orthopedics/orthodontics	Anterior open bite, eruption disturbances, craniofacial anomalies	Non-specific	Clues for the specialist
8	Syndromic signs	Hearing loss, ocular signs, lymphedema, developmental disorders	Absent	Suggestive of syndromic AI
9	Genetic testing	May confirm AI and identify the underlying the genotype	Not routinely indicated	Useful in cases of suspected AI
10	Management of diagnostic uncertainty	Specialist referral before irreversible decision	MIH is likely if typical criteria are present and no evidence supporting AI	Reduces the risk of inappropriate management

Although this framework summarizes the most consistent diagnostic indicators identified in the reviewed literature [8-11,16], it should be interpreted in light of the limitations of the available data.

Limitations of the available data

This review highlights several limitations of the current evidence base. First, the included studies were highly heterogeneous in design, ranging from surveys and reviews to retrospective analyses and molecular case series [12-18]. This heterogeneity limits direct comparison across studies. Second, only a small number of studies directly compared patients with AI and MIH, and some comparative findings were based on limited sample sizes [16]. Third, several diagnostic indicators, such as taurodontism and anterior open bite, may be highly specific when present but lack sufficient sensitivity to rule out AI when absent [6,11,16,17].

In addition, the clinical overlap between severe forms of MIH and hypomineralized AI remains insufficiently quantified. Data on the diagnostic accuracy of approaches combining clinical, radiographic, and genetic assessments in prospective cohorts are also limited. Finally, although genetic testing is increasingly useful, its diagnostic yield remains incomplete, and variants of uncertain significance may complicate interpretation [6].

Perspectives for future research

Future studies should aim to develop and validate standardized diagnostic criteria to differentiate AI from MIH [5,10]. A recent systematic review by Elfrink et al. further emphasized the limited evidence supporting current diagnostic tools and criteria for MIH and highlighted the need for standardized diagnostic approaches, reinforcing the importance of developing robust diagnostic frameworks for developmental enamel defects [19]. Prospective studies comparing clinical examination, radiographic features, family history, and genetic testing in children with enamel defects are particularly needed [6,16]. Further research should also clarify the diagnostic value of taurodontism, anterior open bite, involvement of the primary dentition, and hypomineralization of primary second molars [3,10,11,17]. Given the documented diagnostic uncertainty, there is also a need to evaluate educational interventions targeting general dental practitioners and dental students. Finally, future work should explore whether artificial intelligence-based image analysis, combined with clinical and radiographic data, may help identify complex or ambiguous enamel abnormalities [15,19].

## Conclusions

Differentiating AI from MIH remains a diagnostic challenge in pediatric dentistry, particularly when AI presents in hypomineralized forms that may mimic MIH. This review highlights that a reliable distinction cannot rely solely on the visual appearance of lesions but requires a structured assessment integrating distribution, symmetry, dentition involvement, family history, radiographic findings, syndromic features, and, when indicated, genetic testing. The proposed stepwise diagnostic framework aims to support clinical reasoning, reduce diagnostic uncertainty, and promote specialist referral prior to any irreversible treatment decisions. Prospective studies are needed to validate this framework, clarify the predictive value of its individual criteria, and assess its impact on diagnostic accuracy and treatment planning.
